# Does Improved Survival Lead to a More Fragile Population: Time Trends in Second and Third Hospital Admissions among Men and Women above the Age of 60 in Sweden

**DOI:** 10.1371/journal.pone.0099034

**Published:** 2014-06-09

**Authors:** Korinna Karampampa, Tomas Andersson, Sven Drefahl, Anders Ahlbom, Karin Modig

**Affiliations:** 1 Institute of Environmental Medicine, Unit of Epidemiology, Karolinska Institutet, Stockholm, Sweden; 2 Centre for Occupational and Environmental Medicine, Stockholm County Council, Stockholm, Sweden; 3 Department of Sociology, Demography Unit, Stockholm University, Stockholm, Sweden; INRCA, Italy

## Abstract

**Background:**

Life expectancy and time to first hospitalization have been prolonged, indicating that people live longer without needing hospital care. Life expectancy increased partially due to improved survival from severe diseases, which, however, could lead to a more fragile population. If so, time to a subsequent hospitalization could decrease. Alternatively, the overall trend of improved health could continue after the first hospitalization, prolonging also the time to subsequent hospitalizations. This study analyzes trends in subsequent hospitalizations among Swedish men and women above the age of 60, relating them to first hospitalization. It also looks at trends in the proportion of never hospitalized.

**Methods:**

Individuals were followed in national registers for hospital admissions and deaths between 1972 and 2010. The proportion of never hospitalized individuals at given ages and time points, and the annual change in the risks of first and subsequent hospitalizations, were calculated.

**Findings:**

An increase in the proportion of never hospitalized was seen over time. The risks of first as well as subsequent hospitalizations were reduced by almost 10% per decade for both men and women. Improvements were observed mainly for individuals below the ages of 90 and up to the year 2000.

**Conclusions:**

The reduction in annual risk of both first *and* subsequent hospitalizations up to 90 years of age speaks in favor of a postponement of the overall morbidity among the elderly and provides no support for the hypothesis that the population becomes more fragile due to increased survival from severe diseases.

## Introduction

Due to the increasing life expectancy, the question regarding the health of the elderly has become very important [1], [2]. By the year 2020, 33% of the population in developed countries is projected to be older than 60 years [3], a potential challenge for medical and social care [4].

One way to understand the general health status of the population is to examine time trends in incidence and mortality rates of major diseases, such as cardiovascular diseases (CVD). CVD are major causes of morbidity and death in the older population, and a decrease in both incidence and mortality has been observed since the 1960s [5]–[8]. While such trends provide important insights for specific diseases, they do not necessarily reflect overall health trends. The annual change in hospitalization rates for all diagnoses is an alternative measure that provides information regarding the overall health of the population. The interpretation of such a change, however, is not straightforward since healthcare practices have evolved simultaneously.

In an earlier study we have shown that in parallel with an increase in life expectancy, the mean age at first hospital admission after the age of 60 has been postponed in Sweden by approximately 2 years between 1995 and 2010 [9]. Improved survival from severe diseases explains part of the increased life expectancy and the survivors may constitute a particularly fragile part of the population. This raises the question of whether the time to a subsequent hospitalization decreases. Alternatively, the overall trend of improved health could continue also after the first hospital event in which case one would expect a prolongation also of the time to a subsequent hospitalization.

This study aims to describe trends in subsequent hospitalizations among Swedish men and women above the age of 60, relating them to first hospitalization. It also examines the proportion of individuals never hospitalized in order to provide an overview of the overall health trends in the Swedish population from a novel perspective.

## Materials and Methods

### Study material

Our study population was created from the Total Population Register in Sweden [10]. Information regarding the date of birth and migration status of individuals was collected. In addition, the Total Population Register was used together with the Longitudinal Integration Database for Health Insurance and Labour Market Studies (LISA) [11], which includes yearly information about individuals' income, pensions and social transfers, in order to identify the individual's place of residence from 1972 onwards.

The Patient Register includes individual data regarding all hospital admissions in Sweden; it was used to obtain information about hospitalizations. This register has nation-wide coverage since 1987; however, Stockholm and Uppsala counties have had full coverage in this register since 1972 [12]. To be able to describe hospitalization trends for the longest period possible, we limited our study cohort to these two counties - combined they represent about 20% of the entire Swedish population above the age of 60.

Information on death was obtained from the Cause of Death Register. It includes deaths occurring within or outside Sweden for individuals who were registered in Sweden at the time of death [13].

Information about inpatient care and death was linked to the study cohort using a unique personal identification number that every person residing in Sweden holds. The linking procedure was done by Statistics Sweden and the researchers received a de-identified dataset.

### Setting

All men and women above the age of 60, who were living in the Stockholm and Uppsala counties since 1972, were included in the study population. They were followed from the year 1972 to 2010. The follow-up ended when one of the following events occurred: hospitalization, death, or December 31, 2010. Individuals who moved away from Stockholm and Uppsala counties after 1972 were censored at the time they left.

### Statistical analyses

The outcome under study was hospital admission due to any cause, which had a minimum duration of two nights, and took place after individuals turned 60. The first, second, and third hospital admissions were analyzed, regardless of the outcome being fatal or not. The second and the third hospital admissions had to occur at least 91 days apart from the previous admission.

#### Proportion of non-hospitalized men and women over the period 1972 to 2010

A Kaplan-Meier estimator was used to calculate the proportion of individuals that had never been admitted to the hospital at a given age. Five different birth cohorts, 1912, 1916, 1920, 1924, and 1928 were analyzed and the proportions of non-hospitalized individuals were compared for six ages; 70, 75, 80, 85, 90, and 95 years-old.

#### Hospitalization risks over the period 1972–2010

Using a discrete time logistic model with a complementary *loglog* link [14], the annual relative change in the age-specific annual risk (RR) of being admitted to the hospital for the first, second, and the third time after the age of 60 was estimated. For example, the relative change in the age-specific annual risk for being admitted to the hospital for the first time in 1999 was compared with that of 1998. The average annual relative change over all ages, stratified first in nine different age groups (60–64, 65–69, 70–74, 75–79, 80–84, 85–89, 90–94, 95–99, and 100+), and additionally in four different time periods (1972–1980, 1981–1990, 1991–2000, and 2001–2010) was calculated. Then, the annual change in the risk was estimated by subtracting the relative change from one (1-RR).

For the relative risk of the first admission to the hospital, age at first hospitalization (treated as a categorical variable) was used as a time-varying predictor of the outcome in a regression model. For the second hospital admission, both the age of individuals (treated as a categorical variable), and the time (in years) since the first admission to the hospital, were considered to have an impact on the regression outcome. Therefore an interaction term “*age*×*years since first hospitalization*” was included in the regression model to capture the effect of both parameters simultaneously. The model predictor (time varying) was age at second admission to the hospital. A similar analysis was done for the relative risk of the third hospitalization with age (treated as a categorical variable) being the model predictor, and adjusted for the interaction “*age*×*years since second hospitalization*”.

### Sensitivity analyses

In order to explore the impact of three of the most important causes of death and hospital admissions of older individuals, analyses were made where hospitalizations related to (1) CVD, (2) neoplasms, and (3) mental, behavioral and neurodevelopmental disorders (including dementia and Alzheimer's disease) where removed one at a time. The International Classification of Diseases (ICD) codes were used to identify and exclude the relevant hospital admissions. CVD were defined as; ICD 8 & 9 codes: 390–460, ICD 10 codes: I00-I99. Neoplasms were defined as; ICD 8 & 9 codes: 140–206, ICD 10 codes: C00-C97 and D00-D99. Mental, behavioral and neurodevelopmental disorders were defined as ICD 8&9 codes: 290–319, and 331, ICD 10 codes: F00-F99, and G30.

Additional sensitivity analyses were conducted by altering the minimum duration of hospital stay from two nights to one and three nights respectively. Since there have been changes in the health care policy in Sweden over time affecting the length of stay in hospitals, going from longer and fewer to shorter and more frequent admissions [15], we also tested the minimum transition time from first to second and from second to third hospital admission by running the analysis with 365 days as a minimum time apart between the events.

### Ethical permission

An ethics approval for this study was obtained from the regional ethics committee in Stockholm, Dnr 2011/136-31/5. All databases used in this study were linked using the individuals' personal identification number (*personnumer*). The linkage was conducted by Statistics Sweden and researchers received anonymized data.

## Results

### Proportion of non-hospitalized men and women over the period 1972 to 2010

Tables 1a and 1b present the proportion of individuals without any admission to the hospital at different ages, across different birth cohorts, for men (Table 1a) and women (Table 1b). An increase in the proportion of never hospitalized was seen over time. Even among the 90-year olds, the proportion has increased although the proportion of never hospitalized 90-year olds was rather small (0.7% among men and 1.8% among women). Overall, the proportion of women never hospitalized was smaller compared to men.

**Table 1:** Proportion of men and women at different ages without any admission to hospital.

*A - Men*						
*Birth Cohorts*	*Age 70*	*Age 75*	*Age 80*	*Age 85*	*Age 90*	*Age 95*
1912	28.80%	14.30%	5.80%	2.10%	0.60%	0.10%
1916	28.50%	14.90%	6.10%	2.10%	0.50%	
1920	29.90%	15.90%	7.40%	3.00%	0.70%	
1924	31.20%	18.00%	9.00%	3.40%		
1928	33.10%	19.80%	10.30%			

*B - Women*						
*Birth Cohorts*	*Age 70*	*Age 75*	*Age 80*	*Age 85*	*Age 90*	*Age 95*
1912	42.10%	25.90%	13.70%	5.40%	1.50%	0.20%
1916	39.40%	25.10%	12.90%	5.40%	1.60%	
1920	40.00%	24.90%	13.60%	6.20%	1.80%	
1924	40.10%	25.10%	14.40%	6.60%		
1928	42.00%	28.20%	15.20%			

### Hospitalization risks over the period 1972–2010

In Tables 2a and 2b the annual decrease in the risk of a first, second, and third admission to the hospital between 1972 and 2010 is presented. Results are shown as an average effect for all ages above 60 as well as stratified in nine age groups, for men (Table 2a) and women (Table 2b). In Tables 3a and 3b, results are presented, stratified in four different time periods, for men (Table 3a) and women (Table 3b).

**Table 2:** Annual decrease in the risk of first, second, and third hospital admission after the age of 60, for men and women.

A - Men										
	Average across all ages	age 60–64	age 65–69	age 70–74	age 75–79	age 80–84	age 85–89	age 90–94	age 95–99	age 100 +
First hospital admission, risk+ (95% CI)	0.91%(0.88%,0.94%)	1.20%(1.15%,1.26%)	0.85%(0.78%,0.92%)	0.73%(0.65%,0.81%)	0.71%(0.62%,0.81%)	0.65%(0.52%,0.78%)	0.26%(0.02%,0.5%)	−0.12%(−0.66%,0.42%)	0.48%(−1.02%,1.96%)	1.19%(−4.96%,6.98%)
Second hospital admission, risk+ ^,^ * (95% CI)	0.86%(0.82%,0.91%)	0.73%(0.65%,0.8%)	1.01%(0.92%,1.11%)	1.08%(0.97%,1.2%)	0.91%(0.77%,1.04%)	0.75%(0.57%,0.93%)	0.35%(−0.01%,0.72%)	−0.38%(−1.27%,0.49%)	−1.30%(−4.4%,1.71%)	n.a.n.a.n.a.
Third hospital admission, risk+ ^,^ # (95% CI)	0.77%(0.71%,0.83%)	0.45%(0.33%,0.58%)	0.74%(0.62%,0.86%)	1.19%(1.06%,1.32%)	1.11%(0.97%,1.26%)	0.66%(0.48%,0.85%)	0.05%(−0.26%,0.37%)	−0.32%(−1.05%,0.41%)	−0.78%(−3.54%,1.91%)	−1.72%(−29.81%,20.29%)

B – Women										
	Average across all ages	age 60–64	age 65–69	age 70–74	age 75–79	age 80–84	age 85–89	age 90–94	age 95–99	age 100 +
First hospital admission, risk+ (95% CI)	0.67%(0.64%,0.7%)	0.85%(0.8%,0.9%)	0.60%(0.53%,0.66%)	0.59%(0.52%,0.66%)	0.51%(0.44%,0.59%)	0.82%(0.73%,0.91%)	0.47%(0.32%,0.61%)	−0.21%(−0.51%,0.09%)	−1.44%(−2.26%,−0.62%)	0.14%(−2.7%,2.9%)
Second hospital admission, risk+ ^,^ * (95% CI)	0.73%(0.69%,0.77%)	0.55%(0.48%,0.63%)	0.87%(0.78%,0.96%)	0.98%(0.89%,1.08%)	0.99%(0.88%,1.09%)	0.51%(0.38%,0.63%)	0.05%(−0.16%,0.26%)	−0.47%(−0.93%,−0.01%)	−1.57%(−3.11%,−0.06%)	0.0179(−4.48%,7.69%)
Third hospital admission, risk+ ^,^ # (95% CI)	0.64%(0.59%,0.69%)	0.16%(0.04%,0.29%)	0.76%(0.64%,0.88%)	0.98%(0.87%,1.1%)	1.08%(0.97%,1.2%)	0.53%(0.4%,0.65%)	−0.02%(−0.2%,0.17%)	−0.08%(−0.46%,0.3%)	0.28%(−1.02%,1.56%)	−2.01%(−7.87%,3.53%)

+ annual decrease in the risk of hospitalization. A negative percentage indicates an annual increase in the risk.

*adjusted for the interaction between age and the number of years since the first hospital admission.

# adjusted for interaction between age and the number of years since the second hospital admission.

n.a. measurement not available due to very few observations.

**Table 3:** Annual decrease in the risk of first, second, and third hospital admission after the age of 60, stratified in four different time periods, for men and women.

A - Men												
Period:	1972–1980			1981–1990			1991–2000			2001–2010		
First hospital admission, risk+ (95% CI)	−4%	(−12.3%	,3.69%)	1.53%	(1.25%	,1.81%)	3.38%	(3.09%	,3.67%)	−0.45%	(−0.78%	,−0.11%)
Second hospital admission, risk+ ^,^ * (95% CI)	2.74%	(2.26%	,3.22%)	1.34%	(1.02%	,1.66%)	2.14%	(1.81%	,2.47%)	0.11%	(−0.29%	,0.5%)
Third hospital admission, risk+ ^,^ # (95% CI)	3.56%	(2.88%	,4.25%)	1.29%	(0.91%	,1.67%)	2.01%	(2.01%	,2.01%)	−0.69%	(−1.15%	,−0.23%)

B - Women												
Period:	1972–1980			1981–1990			1991–2000			2001–2010		
First hospital admission, risk+ (95% CI)	−2.70%	(−2.92%	,−2.47%)	2.11%	(1.88%	,2.34%)	1.93%	(1.69%	,2.17%)	−0.44%	(−0.73%	,−0.15%)
Second hospital admission, risk+ ^,^ * (95% CI)	3.04%	(2.63%	,3.45%)	1.76%	(1.5%	,2.03%)	1.51%	(1.24%	,1.78%)	−0.71%	(−1.04%	,−0.38%)
Third hospital admission, risk+ ^,^ # (95% CI)	3.55%	(2.95%	,4.14%)	1.62%	(1.3%	,1.93%)	1.60%	(1.3%	,1.9%)	−0.66%	(−1.03%	,−0.29%)

+ annual decrease in the risk of hospitalization. A negative percentage indicates an annual increase in the risk.

*adjusted for the interaction between age and the number of years since the first hospital admission.

# adjusted for interaction between age and the number of years since the second hospital admission.

The risks of being hospitalized both for the first and the second time after the age of 60 decreased on average by about 9% per decade for men as well as for women. This risk reduction for the first admission to the hospital was observed in almost all age groups - it was higher among the youngest and tended to level off at the highest ages. However, for the second hospital admission, the risk reduction was only observed for men and women up to the age of 89.

For the third admission to the hospital, the reduction in the risk was slightly lower compared to the one estimated for the first and second hospital admission; for men, a reduction of 8% per decade was observed and for women 6%. Among the oldest (90+), an increase in the risk of a third admission to the hospital was observed.

When stratifying the results in four different time periods, for both men and women, a rather high reduction in the risk of subsequent admissions was observed between the years 1972 and 1980 (about 3% per year for the second admission and about 4% per year for the third admission to the hospital). However, results pointed in the opposite direction for the first hospital admission; a 4% increase per year in the risk was observed for men and a 3% increase for women.

Between the years 1980 and 2000, for the first *and* subsequent hospital admissions, an annual risk reduction of 2% was observed for both men and women. During the latest decade, a small annual increase in the risk was observed for the first admission to the hospital for both men and women (0.4% per year), among women with a second hospital admission (0.7% per year), and for both men and women with a third hospital admission (0.7% per year). Among men, no change in the risk of a second admission to the hospital was observed between 2001 and 2010.

### Results from the sensitivity analyses

Excluding CVD, neoplasms, and mental, behavioral and neurodevelopmental disorders, or altering the minimum duration of the admission to the hospital to one and three days respectively, had no impact on the observed trends of the proportion of never hospitalized individuals or the annual change in the risk of first, second, and third hospitalization. Similarly, changing the minimum time between a first and a second hospital admission from 91 to 365 days did not have any impact on the trends. The change in the minimum time between a second and a third admission to the hospital resulted in a slightly lower annual risk reduction for men for the third hospital admission (0.7% reduction instead of 0.8%) while no changes were observed for women.

## Discussion

This study investigated trends in subsequent hospital admissions above the age of 60. In an earlier study, we concluded that individuals nowadays suffer less from illnesses leading to hospitalization [9]. Such a reduction, however, reflects only an initial improvement. Individuals may be more fragile once they have survived the disease that led to the first hospital stay and, hence, subsequent hospitalizations may occur more rapidly. However, our analyses of the change in the risk of subsequent hospital admissions showed no support for this, at least not for ages up to 90 years.

The annual risk for a second hospitalization decreased by almost 10% per decade for ages up to 90 years, a similar improvement as for the risk of first admission. The risk for a third admission to the hospital also decreased but less than the one for the first and second admission. Above age 90, no clear improvement over time was observed for subsequent hospital admissions, which may be a consequence of a more fragile population. On the other hand no worsening was seen either – the risk remained stable.

Since the analyses of changes in the risk of subsequent hospital admissions are based only on the part of the population that has been hospitalized previously, changes in the risk must be interpreted in conjunction with the change in the proportion of the population that has never been hospitalized. An improvement for subsequent hospital admissions alone does not mean necessarily an improvement for the whole population as the proportion of the population never hospitalized could have decreased at the same time. However, during our observation period we observed both a decreasing risk for subsequent hospital admissions and a simultaneous increase in the proportion of never hospitalized – in all ages up to 90.

The finding of a lack of improvement regarding the risk of subsequent admissions among the very oldest, above 90, is in line with a previous study of Swedish centenarians where death rates above age 100 appeared to have been stable between 1969 and 2008 [16], [17], in contrast to younger ages where a continuous reduction has been observed.

This risk reduction for hospitalization was mainly evident during the 1980s and 1990s, and no improvements were seen for the most recent decade (2000–2010). We have no clear explanation to this. It may be a temporal stagnation that will not affect the overall trend when allowing longer follow up in the next decade, or it may be a sign of a real stagnation of improvements. It could also be the effect of a single (period-) factor such as the increase in elective surgery like hip replacement during the past decade [18]. On the other hand, such procedures still constitute a minor part of all hospital admissions in Sweden and therefore the impact on the general trends should be minor.

Even if our results speak in favor of a postponement of morbidity and an amelioration of the overall health of the population, other explanations must be considered as well. The shift of some treatments from inpatient to outpatient care, the availability of home care services to serve the needs of the elderly or other sensitive groups outside a hospital setting [19], and the cut in the number of hospital beds, are factors that need to be considered. A report from the Swedish National Board of Health and Welfare showed a significant reduction in the inpatient care (in favor of outpatient care) received for diseases of the eye and adnexa, asthma, ulcers and inguinal hernia between 1987 and 2010 [20]. However, this shift is unlikely to fully explain the observed trends since only a minority (depending on the disease) of individuals is receiving inpatient care due to these diseases [9].

Home care programs have been designed to enable the elderly to continue an independent lifestyle as opposed to being admitted to the hospital for very long periods, becoming institutionalized in nursing homes and other care facilities [19]. It is therefore possible that such programs would be the underlying mechanism of a shift in second and third hospitalization to higher ages over time – elderly individuals receiving home care may not be admitted to a hospital for conditions that can be treated/monitored through home care services. However, it is difficult to evaluate how much the effect of such programs would be, given that there are other factors as well contributing to the observed trends for the risk of subsequent hospitalizations.

Regarding the cut in number of hospital beds, it is difficult to separate any effect of this on the risk of hospitalization since it is reasonable to believe that the cut is an effect of a more effective health care and a better control of diseases in primary care making the outcome of diseases less severe. It can be observed in the data that over time inpatient care has shifted from longer to shorter and more frequent hospitalizations.

Finally, interpreting our results of a reduction in the risk of both first *and* subsequent hospital admissions, together with findings from other studies showing declining trends in the incidence and mortality of several important diseases among the elderly such as CVD [8], malignancies [21], [22], and dementia [23], lead us to believe that it is conceivable that there has been a postponement of overall severe morbidity in line with the decrease in mortality over the years. Whether these improvements will be extended also to the very oldest, above 90 and 100 years of age, remains to be answered, together with the question of whether less severe morbidity that is treated in primary care has improved as well.

## Conclusions

The risk of a subsequent hospitalization has not increased suggesting that the health of the general population is not worsened due to a higher proportion of people with compromised health that could follow from the improved survival in severe diseases. The lack of improvements in the most recent decade suggests the need for further surveillance of the trends.

## References

[1] Bronnum-Hansen H, Petersen I, Jeune B, Christensen K (2009) Lifetime according to health status among the oldest olds in Denmark. Age and ageing 38: 47–51. 19017677 10.1093/ageing/afn239 PMC2720779

[2] Vaupel JW, Zhang Z, van Raalte AA (2011) Life expectancy and disparity: an international comparison of life table data. BMJ open 1: e000128. 10.1136/bmjopen-2011-000128 PMC3191439 22021770

[3] Rasmussen LJ, Sander M, Wewer UM, Bohr VA (2011) Aging, longevity and health. Mechanisms of ageing and development 132: 522–532. 21820462 10.1016/j.mad.2011.07.004 PMC5167480

[4] Larsson K, Thorslund M (2006) Chapter 8: old people's health. Scandinavian journal of public health Supplement 67: 185–198. 10.1080/14034950600677253 16762910

[5] Cooper R, Cutler J, Desvigne-Nickens P, Fortmann SP, Friedman L, et al (2000) Trends and disparities in coronary heart disease, stroke, and other cardiovascular diseases in the United States: findings of the national conference on cardiovascular disease prevention. Circulation 102: 3137–3147. 11120707 10.1161/01.cir.102.25.3137

[6] Kesteloot H, Sans S, Kromhout D (2006) Dynamics of cardiovascular and all-cause mortality in Western and Eastern Europe between 1970 and 2000. European heart journal 27: 107–113. 16204263 10.1093/eurheartj/ehi511

[7] Levi F, Chatenoud L, Bertuccio P, Lucchini F, Negri E, et al (2009) Mortality from cardiovascular and cerebrovascular diseases in Europe and other areas of the world: an update. European journal of cardiovascular prevention and rehabilitation: official journal of the European Society of Cardiology, Working Groups on Epidemiology & Prevention and Cardiac Rehabilitation and Exercise Physiology 16: 333–350. 10.1097/HJR.0b013e328325d67d 19369880

[8] Modig K, Andersson T, Drefahl S, Ahlbom A (2013) Age-specific trends in morbidity, mortality and case-fatality from cardiovascular disease, myocardial infarction and stroke in advanced age: evaluation in the Swedish population. PloS one 8: e64928. 23741426 10.1371/journal.pone.0064928 PMC3669144

[9] Karampampa K, Drefahl S, Andersson T, Ahlbom A, Modig K (2013) Trends in age at first hospital admission in relation to trends in life expectancy in Swedish men and women above the age of 60. BMJ open 3: e003447. 10.1136/bmjopen-2013-003447 PMC3787478 24065698

[10] Statistics Sweden [Statistika Centralbyrån] (2013) Total Population Register [Registret över totalbefolkningen (RTB)]. Available: http://www.scb.se/Pages/List____257499.asp. Accessed: 31 October, 2013.

[11] Statistics Sweden [Statistika Centralbyrån] (2013) Longitudinal integration database for health insurance and labour market studies (LISA by Swedish acronym). Available from: http://www.scb.se/Pages/List____257743.aspx. Accessed: 31 October, 2013.

[12] National Board of Health and Welfare [Socialstyrelsen] (2013) Patient Register [Patientregistret]. Available: http://www.socialstyrelsen.se/register/halsodataregister/patientregistret. Accessed: 31 October, 2013.

[13] National Board of Health and Welfare [Socialstyrelsen] (2013) Cause of Death Register [Dödsorsaksregistret]. Available: http://www.socialstyrelsen.se/register/dodsorsaksregistret. Accessed: 31 October, 2013.

[14] Modig K, Drefahl S, Ahlbom A (2013) Limitless longevity: Comment on the Contribution of rectangularization to the secular increase of life expectancy. International journal of epidemiology. 10.1093/ije/dyt035 PMC3733697 23657198

[15] Socialstyrelsen [National Board of Health and Welfare] (2012) Patientregistret för 2010 ur ett DRG-perspektiv [National In-patient Care Register in 2010, from a DRG perspective]. Available: http://www.socialstyrelsen.se/publikationer2012/2012-5-25. Accessed: 16 Nov 2012.

[16] Drefahl S, Lundstrom H, Modig K, Ahlbom A (2012) The era of centenarians: mortality of the oldest old in Sweden. Journal of internal medicine 272: 100–102. 22243214 10.1111/j.1365-2796.2012.02518.x

[17] Modig K, Drefahl S, Ahlbom A (2013) Limitless longevity: Comment on the Contribution of rectangularization to the secular increase of life expectancy. International journal of epidemiology 42: 914–916. 23657198 10.1093/ije/dyt035 PMC3733697

[18] Garellick G, Kärrholm J, Rogmark C, Rolfson O, Herberts P (2011) Swedish Hip Arthroplasty Register, Annual Report. Swedish Hip Arthroplasty Register.

[19] Holosko MJ, Holosko DA, Spencer K (2009) Social services in Sweden: an overview of policy issues, devolution, and collaboration. Social work in public health 24: 210–234. 19266401 10.1080/19371910802595299

[20] National Board of Health and Welfare [Socialstyrelsen] (2011) Inpatient diseases in Sweden 1987–2010 [Sjukdomar i sluten vård 1987–2010]. Available: http://www.socialstyrelsen.se/Lists/Artikelkatalog/Attachments/18410/2011-9-5.pdf. Accessed: 16 November, 2012.

[21] Bosetti C, Bertuccio P, Malvezzi M, Levi F, Chatenoud L, et al.. (2013) Cancer mortality in Europe, 2005–2009, and an overview of trends since 1980. Annals of oncology: official journal of the European Society for Medical Oncology/ESMO. 10.1093/annonc/mdt301 23921790

[22] Karim-Kos HE, de Vries E, Soerjomataram I, Lemmens V, Siesling S, et al (2008) Recent trends of cancer in Europe: a combined approach of incidence, survival and mortality for 17 cancer sites since the 1990s. European journal of cancer 44: 1345–1389. 18280139 10.1016/j.ejca.2007.12.015

[23] Qiu C, von Strauss E, Backman L, Winblad B, Fratiglioni L (2013) Twenty-year changes in dementia occurrence suggest decreasing incidence in central Stockholm, Sweden. Neurology 80: 1888–1894. 23596063 10.1212/WNL.0b013e318292a2f9

